# Colorectal cancer screening in African Americans: practice patterns in the United States. Are we doing enough?

**DOI:** 10.1093/gastro/gow005

**Published:** 2016-04-11

**Authors:** Abhijeet Waghray, Alok Jain, Nisheet Waghray

**Affiliations:** ^1^Department of Medicine, MetroHealth Medical Center/Case Western Reserve University, Cleveland, OH, USA; ^2^Division of Gastroenterology and Hepatology, MetroHealth Medical Center/Case Western Reserve University, Cleveland, OH, USA

**Keywords:** colorectal cancer, screening, race

## Abstract

**Background:** Colorectal cancer (CRC) is a common form of malignancy and a leading cause of death in the United States. Screening decreases CRC incidence and mortality. African Americans are at an increased risk of developing CRC, and recommendations are to initiate screening at the age of 45. This study aims to assess the rate of screening for colorectal cancer in African Americans between the ages of 45–49.

**Methods:** African Americans between the ages of 45–49 were identified in the Explorys national database. Patients who completed a colonoscopy, sigmoidoscopy or fecal occult blood test were identified and stratified by sex and insurance status. A *P* value < 0.05 was considered significant.

**Results:** A total of 181 200 African Americans were identified as eligible for screening. Only 31 480 patients (17.4%) received at least one screening procedure for CRC. The majority of patients (66.7%) were screened via colonoscopy. African American females were more likely to complete a screening test (17.8% *vs* 16.7%; *P* < 0.01). The majority of patients (66.0%) who completed a screening test had private insurance.

**Conclusion:** Race, gender and barriers to medical care contribute to disparities in CRC screening rates. Among African Americans, CRC screening remains suboptimal. Tailored public health initiatives, medical record alerts and improved communication between providers and patients are fundamental to addressing issues that impact poor adherence to CRC screening in African Americans.

## Introduction

Colorectal cancer (CRC) remains the third most common malignancy and is a leading cause of cancer-related mortality in the United States [1,2]. The most common pathway to invasive CRC involves progression of premalignant adenomas or serrated polyps over time. This offers a unique opportunity for early detection and treatment [3]. Five-year survival rates are > 90% for patients diagnosed with early-stage CRC compared with < 10% in metastatic disease [4]. Current modalities recommended for screening include colonoscopy, sigmoidoscopy or fecal occult blood testing. While CRC screening initiatives have become widespread, studies demonstrate that adherence with screening recommendations remains poor, with only 40% of CRC cases diagnosed at an early stage [5,6].

The incidence of CRC is highest amongst African Americans compared with other ethnic groups, and mortality is significantly higher compared with Caucasians [7–10]. Several factors have been associated with the increased risk of CRC in African Americans and include diet/nutrition, physical inactivity, smoking, genetic susceptibility and lower use of screening/diagnostic testing [11–13]. Compared with other ethnic groups, a higher proportion of African Americans present with colon cancer prior to the age of 50, more advanced disease at diagnosis and a lower overall five-year survival rate [9,14]. Corroborating these findings, a California-based study demonstrated that 10.6% of African Americans were diagnosed with colon cancer before the age of 50 compared with 5.5% of Caucasians. Risk stratification is an important factor when establishing screening protocols. Based on the early age of onset, increased incidence and mortality, the 2008 CRC screening guidelines by the American College of Gastroenterology (ACG) recommended CRC screening in African Americans beginning at 45 years of age (Grade 2 C) [15]. By comparison, patients at average risk are offered screening beginning at 50 years of age.

Estimates of adherence to CRC screening guidelines in average-risk patients vary by age and race but remain suboptimal. In a 2010 report by the American Cancer Society, only 55% of patients between the ages of 50–64 completed screening with 64% completing screening after the age of 65 [16]. Overall, 62% of Caucasians over the age of 50 completed screening, with a significantly lower percentage of African Americans (56%) completing screening and rates as low as 51% in some states [16]. Given the low adherence to screening recommendations in the general population, it would seem likely that compliance rates would also be low in a subset of patients who require earlier screening. The aim of this study was to assess the percentage of African Americans who completed a screening test for CRC between the ages of 45–49. The patients evaluated from our database provide a diverse sample from the United States.

## Methods

African Americans between the ages of 45–49 were identified in the Explorys database from 2009 to 2015. Each patient had a minimum two-year follow-up in the outpatient setting, and all data were identified in Explorys during the month of December 2015. Patients who completed a screening test for colorectal cancer were identified in Explorys by keyword search (*colonoscopy*: ‘colonoscopy, colonoscopy and biopsy of colon, colonoscopic excision of lesion of large intestine, colonoscopic polypectomy, colonoscopy and excision of mucosa of colon, therapeutic colonoscopy, screening for malignant neoplasm of colon or screening colonoscopy’; *fecal occult blood test*: ‘occult blood screening, screening for occult blood in feces, gastrointestinal occult blood test or guaiac test for occult blood in feces’; *flexible sigmoidoscopy*: ‘flexible fiberoptic sigmoidoscopy or flexible fiberoptic sigmoidoscopy with biopsy’). All procedures were completed between ages of 45–49. Patients with underlying (diagnosed prior to the age of 45) intestinal polyposis syndromes, colonic cancer (ICD 9: 153.1, 153.2, 153.3, 153.6, 209.13, 209.14, 209.15, 209.16, 209.53, 209.54, 209.55, 209.56), ulcerative colitis (ICD 9: 556.xx) or Crohn's disease (ICD 9: 555.xx) were excluded from the study. The primary endpoint was the percentage of African Americans who completed a screening test for CRC between the ages of 45–49. When calculating overall screening completion rates for the primary endpoint, patients who received a flexible sigmoidoscopy or a fecal occult blood test followed by colonoscopy were counted as a single screening test. Demographic data for sex and insurance status were reported. Patients with multiple insurance plans, changes in insurance status or with forms of insurance other than Medicaid, Medicare or private insurance were categorized as other/unknown.

Explorys (Explorys Inc, Cleveland Ohio, USA) is a database that contains >44 million patients from 360 hospitals and >25 healthcare networks across the United States. Health information systems such as electronic health records, laboratory and billing systems are used to compile the data in Explorys. Records are de-identified and meet Health Insurance Portability and Accountability Act (HIPAA) and Health Information Technology for Economic and Clinical Health (HITECH) Act standards. Institutional Review Board (IRB) approval was not required.

### Statistical analysis

Patients were divided into two groups: those who were eligible for CRC screening and those who completed at least one test for CRC screening. Demographic data are represented as frequencies and percentages with quantitative variables summarized as means. Categorical data were compared with Fisher's exact test. A *P* value < 0.05 was considered significant, and data were analyzed using JMP version 9.0 (SAS Institute Inc, Cary, NC, USA).

## Results

A total of 181 200 African American patients were identified as eligible for CRC screening. Complete demographic details are listed in Table 1. Among them, 31 480 patients (17.4%) received at least one screening test (colonoscopy, occult blood testing, flexible sigmoidoscopy) between the ages of 45–49. The majority of patients were screened by colonoscopy (*N*=21 010; 66.7%) with 9740 patients (30.9%) screened by fecal occult blood test and 730 patients (2.3%) screened by flexible sigmoidoscopy. Gender differences in CRC screening demonstrated that female patients were more likely to complete at least one screening test (17.8% *vs* 16.7%; *P* < 0.01). Fewer males completed a colonoscopy (10.6% *vs* 12.2%, *P* < 0.01) and opted for less invasive screening methods (FOBT and flexible sigmoidoscopy) (*P* < 0.01) (Table 2). The majority of patients who completed CRC screening had private insurance (*N* = 18 450; 58.6%), while coverage via Medicare and Medicaid accounted for 7.2% (*N* = 2260) and 9.1% (*N* = 2880) of patients, respectively. Further, privately insured patients were more likely to complete CRC screening tests (*P* < 0.01) (Table 3).

**Table:** **Table 1.** Demographic data for patients eligible for colorectal cancer screening

Total	181 200
Sex, n (%)	
Male	71 080 (39.2)
Female	110 120 (60.8)
Insurance status, n (%)	
Private	100 930 (55.7)
Medicaid	16 820 (9.3)
Medicare	10 360 (5.7)
Other/unknown	53 090 (29.3)

**Table 2.** Comparison of screening completion rates between males and females

Screening test	Male (N = 71 080)	Female (N = 110 120)	*P* value
Colonoscopy, n (%)	7570 (10.6)	13 440 (12.2)	<0.01
Fecal occult blood testing, n (%)	4020 (5.7)	5720 (5.2)	<0.01
Flexible sigmoidoscopy, n (%)	280 (0.4)	450 (0.4)	0.65
Total, n (%)	11 870 (16.7)	19 610 (17.8)	<0.01

**Table 3.** Insurance status of patients receiving colorectal cancer screening

Screening test	Private	Medicaid	Medicare	Other/unknown
Colonoscopy, n (%)	13 870 (66.0)	1670 (7.9)	1440 (6.9)	4030 (19.2)
Fecal occult blood testing, n (%)	4240 (43.5)	1160 (11.9)	750 (7.7)	3590 (36.9)
Flexible sigmoidoscopy, n (%)	340 (46.6)	50 (6.8)	70 (9.6)	270 (37.0)

## Discussion

In recent years, increased emphasis has been placed on public education programs for CRC screening. The use of public service announcements, colon cancer awareness month and social media campaigns has resulted in a 6% increase in screening rates for patients over the age of 50 [16,17]. While this trend is promising, overall CRC screening in the general population remains suboptimal. African Americans, in particular, are at increased risk of advanced proximal lesions leading to a higher prevalence of right-sided cancers and overall rates of CRC [18–20]. Despite recommendations for screening to begin at age 45, current programs emphasizing CRC screening at age 50 for average risk patients leave most African Americans unaware of the need for earlier exams. Limited data exist on CRC screening rates in this at-risk population, and to our knowledge no study has assessed the national rates of CRC screening in African Americans between the ages of 45–49.

Based on our study, only 17.4% of African Americans between ages of 45–49 received CRC screening. Approximately 33% of patients in this study were screened via fecal occult testing or flexible sigmoidoscopy. Compared with other ethnic groups, African Americans have a higher prevalence of right-sided CRC, and these proximal lesions would be missed with sigmoidoscopy alone as a screening tool [21–24]. Furthermore, the validity of occult testing as a screening test for CRC is dependent on appropriate administration. A national survey of >1100 primary care physicians revealed that 33% of physicians used the less-accurate single-sample in-office testing rather than the recommended home-based screening method. Compounding this issue, the workup of positive occult testing significantly deviated from established guidelines, with repeat occult testing being recommended by 30% of physicians [25]. While occult testing is a valid method of screening, proper training for the recommended practices and appropriate follow-up of abnormal testing remain an issue.

Barriers to appropriate care confound underlying racial and sex disparities in CRC screening. Availability and access to care remain important factors impacting compliance with CRC screening. Patients in urban settings are more likely to complete CRC screening compared with patients in rural settings, with the largest disparity being among residents in remote rural areas [26]. In one study, about 65% of patients were willing to drive ≤ 30 minutes for a colonoscopy, with the majority of patients (86%) more likely to complete a colonoscopy if a facility were closer to their home [27]. Thus, access to care is pivotal to increasing compliance rates in CRC screening, particularly for those living in rural areas.

As previously mentioned, the emphasis placed on population-based screening at the age of 50 may have implications for African Americans who require earlier preventive care. Other factors impacting the rate of CRC screening include cost and access to healthcare. Over $12 billion is spent annually for treatment of CRC in the United States [28]. The goal of preventative services involves early detection, thereby reducing the economic and healthcare related costs of more advanced disease. Therefore, coverage of screening colonoscopy via the Affordable Care Act (ACA) or other private insurance is prudent. Yet, 16.1% of patients in a recent survey reported that a colonoscopy was a financial strain [29]. Inconsistencies in insurer-defined screening services and coding practices by healthcare providers contribute to unexpected out-of-pocket expenses. Financial liability remains an important deterrent for patients completing screening procedures. This was corroborated by the National Colorectal Screening Network, a survey of healthcare professionals in which 80% of respondents were aware of issues related with insurance coverage and unexpected cost-sharing for screening colonoscopies under the ACA prevention benefits [30,31]. Seventy percent of respondents believed the potential for cost sharing would discourage patients from pursuing screening procedures. While some insurers have waived cost sharing for all colonoscopy procedures, more needs to be done to make the system more economical for patients.

An important factor in the success of any screening program remains effective communication between healthcare providers and patients. Conveying the importance of CRC screening, including the risks and benefits of each modality, is fundamental to the success of screening initiatives. It is well documented that physician involvement in screening programs is crucial for patient participation [32–34]. Limited proficiency in English, inadequate communication by providers and lower education levels all contribute to lower CRC screening [35,36]. Specifically, a survey of African American patients reported limited understanding of CRC, which was associated with a perception that screening was unwarranted [37]. Previous national studies have also shown that amongst African Americans, overall awareness of CRC and the benefits of screening were much lower when compared with their Caucasian counterparts [38–41]. Personal barriers such as fear of CRC screening, cancer diagnosis and perceived embarrassment from invasive procedures also contribute to lower rates of screening in African Americans [32,42]. Further, the “legacy of Tuskegee” has led to issues of distrust towards healthcare providers and overall reservations about visiting healthcare facilities or undergoing invasive procedures [43]. With improved communication and explanation regarding the procedure, it has been shown that African Americans have reported an increased willingness to complete CRC screening [43]. Therefore, communication and education remain key components for healthcare providers to improve CRC screening rates in this at-risk population.

There are several clinical implications for our study. Strategies to increase CRC screening should be targeted towards both patients and healthcare providers. The use of automated electronic or mailed reminders may assist patients in adhering to screening guidelines. Electronic Medical Record (EMR) alerts can also assist healthcare providers to address screening requirements and eliminate uncertainty about recommendations or guidelines. The use of EMR has been successfully implemented to increase vaccination rates and achieve performance measures in chronic diseases [44,45]. Further, nurses, advanced nurse practitioners, primary care physicians and specialists have a unique opportunity to provide a patient-centric approach to discussing screening for CRC. Addressing any concerns patients may have regarding screening recommendations and the risks/benefits of each modality will help to promote awareness and the implementation of a successful CRC screening program. Supporting this approach, a study conducted in a predominantly minority population focused on improving access to CRC screening with patient navigators and an enhanced referral system, resulting in an increased number of patients completing their screening colonoscopies [46]. While emerging noninvasive screening tests such as fecal immunochemical or stool DNA testing may be an option for initial screening, patients must be aware that like fecal occult testing, abnormal results will require endoscopic evaluation. The cost effectiveness of these newer modalities compared with colonoscopy and their role in specific patient populations remains uncertain [47,48].

There are limitations to our study. This is a retrospective study using diagnostic coding that cannot be validated at the individual patient level. Further, limitations of the database do not allow us to assess physician intent to screen *vs* patient non-compliance. Incomplete or suboptimal preparation for colonoscopies and inappropriate use of fecal occult testing cannot be identified. Thus, our study likely overestimates the rate of CRC screening irrespective of the modality utilized. Despite the above limitations, this study involved patients from a cross section of the United States and provides a comprehensive assessment of CRC screening rates in African Americans between the ages of 45-49.

In summary, this study demonstrates that CRC screening rates for African Americans between the ages of 45-49 remains suboptimal. Subgroup analysis revealed that women and individuals with private insurance were more likely to complete screening recommendations for CRC. Successful public health initiatives, EMR alerts and communication between providers and patients are fundamental to addressing issues impacting poor adherence to CRC screening in African Americans. This study clearly demonstrates that more needs to be to done to promote preventative CRC services in the African American community. 

*Conflict of interest statement:* none declared.
